# Steroid hormones: risk and resilience in women’s Alzheimer disease

**DOI:** 10.3389/fnagi.2023.1159435

**Published:** 2023-06-16

**Authors:** Noelia Calvo, Gillian Einstein

**Affiliations:** ^1^Department of Psychology, University of Toronto, Toronto, ON, Canada; ^2^Rotman Research Institute, Baycrest Health Sciences, Toronto, ON, Canada; ^3^Tema Genus, Linköping University, Linköping, Sweden; ^4^Women’s College Research Institute, Toronto, ON, Canada; ^5^Centre for Life Course and Aging, University of Toronto, Toronto, ON, Canada

**Keywords:** steroid hormones, resilience, Alzheimer’s disease, macroglia, microglia, BDNF, plasticity, verbal memory

## Abstract

More women have Alzheimer disease (AD) than men, but the reasons for this phenomenon are still unknown. Including women in clinical research and studying their biology is key to understand not just their increased risk but also their resilience against the disease. In this sense, women are more affected by AD than men, but their reserve or resilience mechanisms might delay symptom onset. The aim of this review was to explore what is known about mechanisms underlying women’s risk and resilience in AD and identify emerging themes in this area that merit further research. We conducted a review of studies analyzing molecular mechanisms that may induce neuroplasticity in women, as well as cognitive and brain reserve. We also analyzed how the loss of steroid hormones in aging may be linked to AD. We included empirical studies with human and animal models, literature reviews as well as meta-analyses. Our search identified the importance of 17-b-estradiol (E2) as a mechanism driving cognitive and brain reserve in women. More broadly, our analysis revealed the following emerging perspectives: (1) the importance of steroid hormones and their effects on both neurons and glia for the study of risk and resilience in AD, (2) E2’s crucial role in women’s brain reserve, (3) women’s verbal memory advantage as a cognitive reserve factor, and (4) E2’s potential role in linguistic experiences such as multilingualism and hearing loss. Future directions for research include analyzing the reserve mechanisms of steroid hormones on neuronal and glial plasticity, as well as identifying the links between steroid hormone loss in aging and risk for AD.

## 1. Introduction

Although abundant research has documented sex disparities in Alzheimer’s disease (AD; World Health Organization, 2021), the sources of these sex differences remain disputed. Women live longer than men and this has been suggested as one of the reasons. Indeed, old age is the most common risk factor for AD, but age alone cannot explain the fact that two-thirds of the AD global cases are women (Snyder et al., 2016). There are likely other factors involved such as sex chromosomes, steroid hormones, brain structure differences, and varying life experiences. Moreover, there are several forms of AD, and they may affect women and men differently (Snyder et al., 2016). AD can be classified into early (≤65 years) or late, familial-sporadic (>65 years) onset. Early onset AD is rare (only 5% of all AD cases) and has a genetic background. Late onset AD represents most AD cases and has a less clear background (Bekris et al., 2010).

Consider the first case of AD identified which we would now call early onset – that of a 51-year-old woman named Auguste Deter. In 1906, Dr. Alois Alzheimer noticed that Auguste had abnormal behaviors such as short-term amnestic problems, disorientation, and dysphasia. He diagnosed the case as ‘presenile dementia’ and later brain biopsy showed senile plaques, neurofibrillary tangles, and atherosclerotic alteration. Since then, numerous studies have confirmed this constellation of signs as AD and the disease has become one of the most intractable, worldwide problems in medicine. AD is complex, there are different subgroups throughout the lifespan (Lima et al., 2022), and its trajectory and phenotypes are typically influenced by multiple pathological processes such as α-synucleinopathologies, vascular pathology, non-AD-tauopathies, etc. (Dubois et al., 2021). Despite its complexity, it is crucial to understand sex differences because biological sex may have a distinctive role in the cause and trajectory of AD.

In this respect, postmortem studies have shown that women have more severe neurodegeneration than men (Filon et al., 2016); and transgenic animal models of AD-like brain amyloidosis have found that female mice have increased plaque load burden and higher levels of soluble and insoluble Aβ40/Aβ42 than age-matched males (Callahan et al., 2001). In humans, it was initially thought that apolipoprotein ε4 (*APOE ε4*) may place women at higher risk than it does men (Altmann et al., 2014). However, a meta-analysis with data on nearly 60.000 participants revealed that only women with *APOE ε4* who are younger than 55 are at increased risk of developing AD (Neu et al., 2017). Recent evidence suggests that sex differences in AD may be more strongly related to regional tau pathology (Buckley et al., 2020) with a longitudinal PET study revealing that women have an increased accumulation rate of tau pathology, even when adjusting for baseline tau load (Smith et al., 2020). These findings demonstrate that clinical research needs to explore sex differences in order to discover specific pathways to AD (e.g., AD tauopathies vs. non-tauopathies, early vs. late onset, etc.).

Indeed, these studies have focused on only some of the theories that explain the cause of AD such as beta amyloid and tau, but female sex has a crucial role in most theories about AD. For instance, the mitochondrial cascade hypothesis posits that mitochondrial DNA (mtDNA) affects a person’s risk of AD (Swerdlow et al., 2014), in this case mothers would have a bigger contribution to risk in their offspring, as mtDNA is maternally inherited. Moreover, estradiol loss due to menopause leads to a reduced mitochondrial function (Scheyer et al., 2018); which in turn may lead to cognitive impairment (van der Windt et al., 2012). Alternatively, AD may be the result of mixed disease pathology given multiple co-pathologies are present in AD patients. A typical AD co-pathology is vascular (Pasquier et al., 1998) as neurovascular dysfunction contributes to cognitive decline (Tarantini et al., 2017). Importantly, neurovascular function is influenced by gonadal hormones (Honarpisheh and McCullough, 2019); in particular, E2 improves blood flow (Collins et al., 1995). In this sense, the decrease in E2 production at menopause is a known risk factor for women’s cardiovascular disease (Vogel et al., 2021). Moreover, the presynaptic protein α-synuclein (αSyn), mainly associated with synucleinopathies may be involved in the pathophysiology of AD. For instance, dementia with Lewy bodies (DLB) is one of the disorders referred to as a-synucleinopathies and, while the disease is more prevalent in men (Kane et al., 2018), once manifested, it has a more aggressive course in women (Van de Beek et al., 2020). Post-mortem examinations reveal that women generally present mixed pathology (DLB + AD) while men show ‘pure’ DLB pathology (Barnes et al., 2019; Van de Beek et al., 2020).

If there are possible sex differences in risk, cause, and trajectory of AD, it is intuitive that there might also be sex differences in resilience–a general term used to refer to several mechanisms that increase reserve and maintain function in the face of AD pathology (Stern et al., 2020). There is both cognitive and brain reserve. Individuals with high cognitive reserve show better than expected cognitive performance given the degree of pathology (Stern, 2002). Brain reserve is the neurobiological state of the brain, such as number of neurons. It can be measured by the connectivity, response, and structural integrity of key brain areas like the hippocampus. Brain maintenance, another type of resilience, refers to reduced age-related brain changes over time based on biological factors or lifestyle; factors that lead to brain maintenance can add to brain reserve (Stern et al., 2020). Since women live longer than men, it is important to consider these resilience mechanisms that are also involved in women’s aging. Specifically, there might be sex differences in resilience related to steroid hormones and sex chromosomes. For instance, a second X chromosome may offer resilience to women (Davis et al., 2020) and steroid hormones might also influence plasticity (Jacobs and Goldstein, 2018) shaping women’s cognitive and brain reserve.

The first report of resilience, while it did not analyze data by sex, was a study with a cohort that was 81% women. The authors examined post-mortem brains of 137 older adults and found extensive AD pathology in some of these patients, but also greater number of neurons, and higher brain weight when compared to age matched controls (Katzman et al., 1988). When considering this study from the perspective of brain reserve, one can imagine that these patients might have had some resilience mechanism that helped them to avoid neuron loss. An example of cognitive reserve comes from one of the most famous longitudinal studies of aging, a cohort of all women (Snowdon, 1997). The authors studied the written autobiographies of 678 novitiate nuns from the Notre Dame congregation, using linguistic density (ability to produce complex human communication) to predict resilience. The results showed that early life texts lacking linguistic density were correlated with increased late-life AD. This was one of the first studies using verbal processing to measure resilience. Nowadays, a verbal advantage is suggested as a form of cognitive reserve in women (Sundermann et al., 2016), but the underlying mechanisms are still not clear.

The purpose of the present review is to explore the possible mechanisms underlying risk and resilience in women especially as they might be mediated by steroid hormones. Note that we limited our searches to studies considering biological sex. The Canadian Institute of health research (CIHR) defines sex as female or male but recognizing variation in the biological aspects that comprise sex and how those aspects are expressed. Gender, on the other hand, refers to the socially constructed roles, behaviors, and expressions of gender diverse people. In this manuscript, we focus on sex instead of gender because we review articles that have investigated biological aspects (e.g., sex hormones, chromosomes) related to risk and resilience. First, we review the role of steroid hormones loss in aging as risk factor for AD. Then, we review the evidence that steroid hormones play a role in the plasticity of neuronal as well as nonneuronal brain cells considering plasticity’s contribution to resilience. We also focus on other possible factors that may further confer cognitive and brain reserve against AD in women. Finally, we highlight existing gaps in the literature, and new avenues for future research.

## 2. Literature search

**Figure 1:**
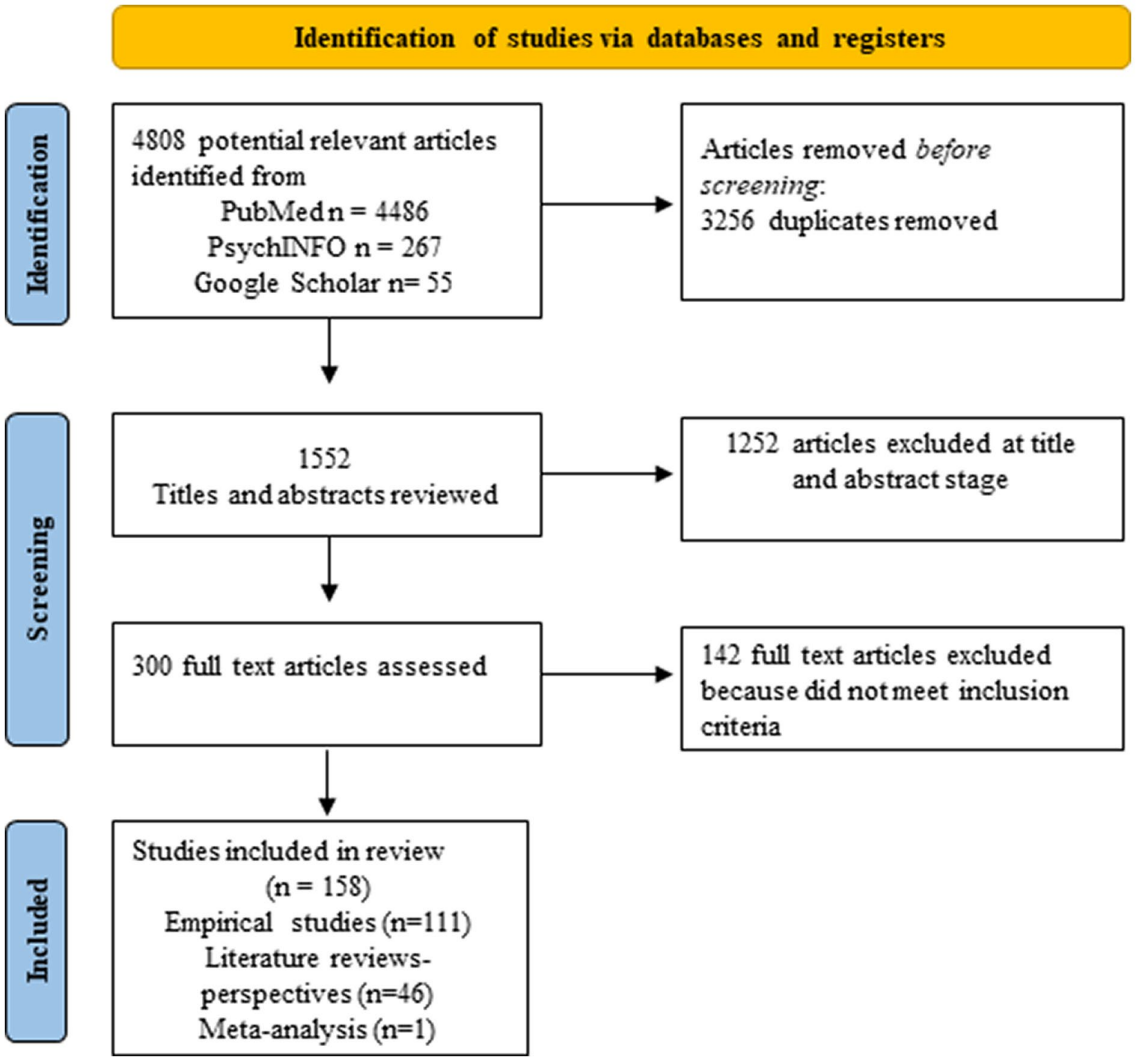
PRISMA flow diagram of the study selection process. The diagram describes all articles included in section 3.

We undertook a wide search of the literature in order to understand the interconnectedness of steroid hormones, risk for AD, plasticity, and resilience. To do this, we used a combination of all the following key words or phrases for the search: aging, AD, brain plasticity, steroid hormones, sex differences, glial cells, estrogens, estradiol, estrogen replacement therapy, cognitive reserve, brain reserve, verbal memory in women, and linguistic experience. Main searches were conducted using PubMed, and PsychINFO databases. A manual search of the reference lists of included studies was also used to identify relevant articles–Google Scholar was used for these specific searches. Articles published between 1990 and April 2023 were included. We included articles starting in 1990 because the National Institutes of Health (NIH) Revitalization Act that required the inclusion of women in NIH-funded clinical research was done in 1993.We conducted a synthetic review of the literature and followed PRISMA guidelines for the selection of articles. We then organized articles into themes and subthemes to make results clearer. This combined approach allows for the integration of many different types of articles, the creation of new theoretical perspectives, the identification of gaps in the literature and the implementation of rigorous methodology for the selection of articles. Thus, we included empirical studies with human and animal models, literature reviews as well as meta-analyses to enhance our knowledge of the literature and be able to identify new emerging perspectives. Inclusion criteria were: 1990 and later, articles that were written in English. Exclusion criteria were: articles primarily focused on neurodegenerative diseases other than AD (e.g., Huntington), neuronal or nonneuronal associations with dementias other than AD, and studies focusing on gender instead of biological sex. We also excluded gray literature such as theses or conference proceedings. Initially, a total of 4,808 articles were identified as potentially relevant, but after screening only 158 full articles were selected (see Figure 1). Note that we also drew on foundational papers that were not part of the search – such as Hebb (1949)–but that formed the conceptual foundation of our questions. Where these are used, they are marked with ^+^ to designate that they were not part of the search. Moreover, our search led to the referencing of other disorders – such as fronto-temporal dementia (FTD); strictly speaking these are not part of the results but we do address them in the discussion.

**Figure 2:**
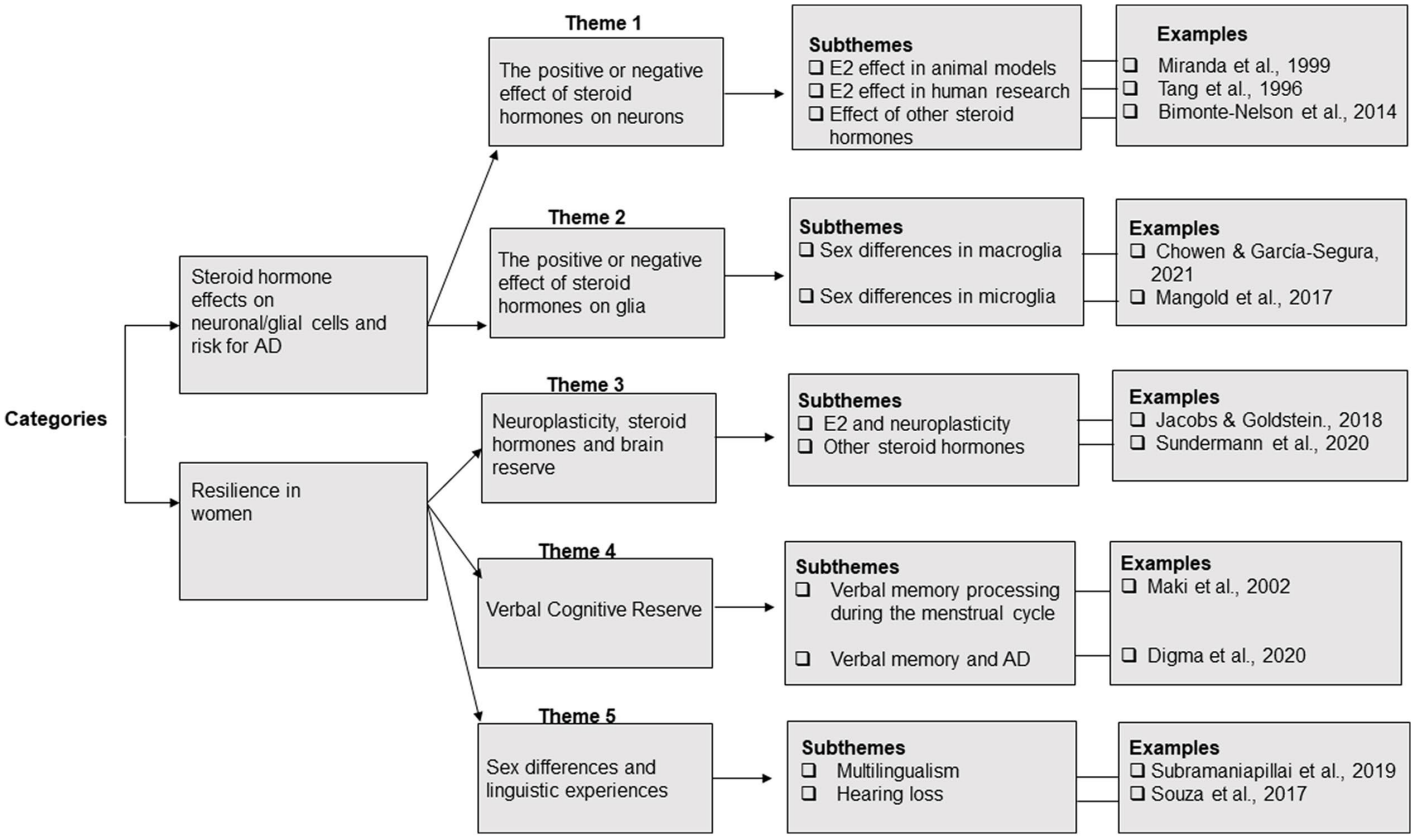
Overview of the analytic process and the categories, themes and subthemes that emerged from the analysis.

We extracted data for all 158 studies. We then entered these data into an Excel file with the following headers: data source, study title, methods and main findings. Based on the full-article texts, the articles were divided into 2 categories: (1) Steroid hormone effects on neuronal/glial cells and risk for AD, and (2) Resilience in women. These categories were not mutually exclusive–meaning that articles addressing risk could also address resilience. We further evaluated articles in each category and identified main themes. To do this, we looked for ideas discussed across and in most articles. Thus, each article was assigned to at least one of the following main themes: (a) the positive or negative effect of steroid hormones on neurons, (b) the positive or negative effect of steroid hormones on glia, (c) neuroplasticity, steroid hormones and brain reserve, (d) verbal cognitive reserve, (e) sex differences and linguistic experience (Figure 2). A narrative synthesis method was used to describe the results.

## 3. Results

### 3.1. Steroid hormone effects on neuronal/glial cells and risk for AD

The average adult brain contains approximately 100 billion cells including neurons and glia cells (nonneuronal). Both neurons and glia are influenced by progesterone, estrogens, and androgens (testosterone) (Chowen and Garcia-Segura, 2021). These steroid hormones modulate neural activity, synaptic plasticity, growth factor expression, neuronal survival (Pike et al., 2009), and cognition (Sherwin, 1997; Boyle et al., 2021). Steroid hormones also interact with certain genes located on the X and the Y chromosomes (Raznahan and Disteche, 2021), and they affect glial cells (Mellon and Vaudry, 2001; Garcia-Ovejero et al., 2005). Recent evidence indicates that steroid hormones may protect the brain against AD pathology (Pike et al., 2009; Vegeto et al., 2020); and thus may explain the sex differences in AD (Einstein, 1999; Nebel et al., 2018; Vegeto et al., 2020; Guo et al., 2022). However, most of the evidence comes from studies analyzing steroid action on neurons, showing sex specific patterns of disease manifestation and differences in the rates of cognitive decline and pathology (for a review see Ferretti et al., 2018). The question of how glia cells contribute to sex differences in AD is less clear. The focus of this section is to review what is known about the impact of steroid hormones at the cellular level in both neurons and glia to better understand how they might contribute to sex differences in risk for AD.

#### 3.1.1. The positive or negative effect of steroids hormones on neurons

Estrogens (estradiol, estriol and estrone), pregnanes (progesterone and allopregnanolone) and androgens (testosterone and dihydrotestosterone) are steroidal hormones produced by the gonads, adrenal glands and other tissues; perhaps most importantly, the brain (Ruiz-Cortés, 2012). All steroid hormones are present in both biological sexes, but at different levels (Miller et al., 2017). There are multiple and different receptors for estrogens (ER, subtypes α and β), progesterone (PRs, subtypes A and B), and androgens (AR). These receptor’s interaction with steroid hormones can affect the nervous system directly or indirectly(Ruiz-Cortés, 2012).

For the most part, research exploring the mechanisms underlying sex differences in AD have focused on the effects of E2 on neurons. For instance, in an animal study, Miranda et al. (1999) showed sex differences in estrogen deprivation on aged rodent dentate granule cells. The authors deprived and restored estrogens to young and old gonadectomized female and male rats and explored whether the secretion of gonadal steroids led to morphological changes to neurons in the hippocampus–an important brain region for memory and learning and affected early in AD (Mielke et al., 2012). Results showed that dentate granule cells of estradiol-deprived female rats had lower spine density than that of testosterone-deprived males or young females with short term estrogen deprivation. Similar effects were reported in female macaques after surgical menopause, showing spine density loss in multiple cortical regions including hippocampal CA1 neurons (Dumitriu et al., 2010). In humans, steroid patterns were reported for postmenopausal women in a study exploring the effects of estrogen in AD. In this case, the authors studied estrogen use in a cohort of 1,124 elderly women in a longitudinal study of aging. E2 use was associated with a delay in the onset as well as a lower risk for AD (Tang et al., 1996). However, the question of hormone replacement therapy (HRT) for older women and in AD is a controversial one. The hypothesis behind this question is that the use of exogenous hormones (e.g., E2) may ameliorate cognitive decline in menopause. The question has critical implications for clinical treatment but human research has reported mixed findings.

Several studies replicated the findings by Tang et al. (1996) using prospective approaches (Baldereschi et al., 1998; Jacobs et al., 1998; Zandi et al., 2002; Shao et al., 2012), cross-sectional (Steffens et al., 1999; Lau et al., 2010), and case control studies (Henderson et al., 1994, 2005). Indeed, a recent meta-analysis including studies using different approaches (prospective, retrospective, etc.) confirmed the benefits of postmenopausal use of E2 and concluded that it significantly decreased the risk of onset and/or development of AD (Song et al., 2020). This meta-analysis; however, did not include the largest randomized clinical trial linking HRT to AD which found the opposite effect. The Women’s Health Initiative (WHI; Shumaker et al., 2003) was a complex study exploring strategies for the prevention of heart diseases in postmenopausal women. In brief, the results showed that HRT did not benefit and might even be detrimental with respect to heart disease, stroke, blood clots, breast cancer and cognitive decline. Notably, the Women’s Health Initiative Memory Study (WHIMS; Shumaker et al., 1998, 2004) replicated these findings–specifically for cognition. Taken together, these studies led to ceasing recommending conjugated equine estrogens (CEE) plus medroxyprogesterone acetate (MPA) to postmenopausal women. Debate about the methodology and cohort of the WHI and its ancillary studies (WHIMS) suggested that these studies were not assessing prevention as most of the women included in the cohort were many years past menopause (Lobo, 2013). The question of the benefits of estrogen replacement therapy (ERT as opposed to CEE) still needs answering because E2 loss after menopause has been correlated with cognitive decline and hence, risk for AD. Also, the question of when hormone replacement begins is important. A recent study found that earlier age at menopause and late initiation of HRT were significantly associated with increased regional tau in the context of high β-amyloid (Coughlan et al., 2023). If women began HRT exactly at the onset of menopause, they did not have higher tau proteins in the brain. However, AD markers were elevated if they delayed the treatment to more than 5 years after menopause.

The possible positive effects of estrogens can also be studied in women who have had bilateral salpingo-oophorectomy (BSO) which leads to a rapid and deep decrease level of E2; they show increased risk of cognitive impairment in late life (Rocca et al., 2007) as well as up to 5 years post-BSO (Gervais et al., 2020), sleep disturbances (Kaunitz et al., 2021), and increased inflammation (Au et al., 2016). Women who underwent BSO before 50 years old have shown smaller amygdala volumes, thinner parahippocampal-entorhinal cortices, and lower entorhinal white matter fractional anisotropy values than women who kept their ovaries (Zeydan et al., 2019). Women with BSO before spontaneous menopause also have an increased risk of MCI and decreased performance on global cognition, attention, executive functions, and short Mental status test (Rocca et al., 2021). Taken together, these findings show that early life BSO is a risk factor for AD which highlights the importance of understanding the unique effects of each menopause and steroid hormones loss (Edwards et al., 2019). There is a more generally acknowledged importance of E2 production in staving off cognitive decline and eventual AD (Pike, 1999; Koski et al., 2004; Quinn et al., 2022) with proposed mechanisms including inducing and maintaining hippocampal spine density (Woolley and McEwen, 1994; Miranda et al., 1999; Leranth et al., 2002; Wallace et al., 2006; Hajszan and Leranth, 2010; Bowman et al., 2015) as well as by controlling β-amyloid accumulation (Gandy and Petanceska, 2000; Qin et al., 2020).

Progesterone as well as estrogens regulate brain-derived neurotrophic factor (BDNF) which influences survival, growth, and maintenance of neurons (Chan and Ye, 2017), and thus the effects of progesterone should also be studied. In animal studies, progesterone has been linked to the survival rate of newborn hippocampal neurons in rats (Deniselle et al., 2007). Progesterone also has a neuroprotective effect, but it seems to be E2-dependent as it reduces E2-induced BDNF (Bimonte-Nelson et al., 2004). More specifically, long-term progesterone treatment in aged ovariectomized female rats has been found to block the beneficial effects of E2 on neurotrophic factor, nerve growth factor, and neurotrophin 3 (Bimonte-Nelson et al., 2004). Thus, the effects of progesterone on neurons should be carefully analyzed considering its interaction with estrogens. Indeed, the few studies that have been conducted in non-human animals indicate that progesterone alone may have some benefits on neuronal activity, but it may also have an antagonistic effect when combined with estrogens (Pike et al., 2009). Understanding the interaction between estrogens and progesterone is crucial because it may lead to a more accurate interpretation of research and clinical trials. For instance, the WHI trial mentioned above reported that CEE therapy leads to an increased risk of AD, but current empirical work suggests that interactions with progesterone may have obscured estrogen’s effects on AD in that study (Pike et al., 2009).

Testosterone is important to consider as it, too, may regulate BDNF (Gao et al., 2019). In non-human animals, androgens reduce neurite outgrowth (Zelleroth et al., 2021), but they may also promote neuron viability (Creta et al., 2010), and offer protection against apoptosis in hippocampal neurons (Nguyen et al., 2010). Humans with prostate cancer treated by androgen deprivation have an increased risk for AD (Seruga and Tannock, 2008). Thus, androgens may also play a contributing role in supporting neurons against AD degeneration. In both women and men, low testosterone may increase the risk of AD. However, only a few studies have explored testosterone as an AD therapy; unsurprisingly, most of these studies are in men (Pike et al., 2009). To our knowledge, no study has explored the effects of testosterone therapy or its interaction with E2 therapy for AD in women.

#### 3.1.2. The positive or negative effect of steroid hormones on glia

Glial cells include macroglia (oligodendrocytes and astrocytes) and microglia; together these non-neuronal cells transport nutrients and carry out other metabolic processes, influence synaptic communication, protect neurons from oxidative stress, and modulate neuronal activity (Heneka et al., 2010; Chowen and Garcia-Segura, 2021). In particular, oligodendrocytes provide insulation through the production of myelin, allowing for high-speed transmission of signals. They support and maintain the integrity of axons (Larson, 2018). Oligodendrocyte function depends on both astrocytes and microglia. Indeed, oligodendrocytes connect intracellularly with astrocytes to aid in the removal of myelin debris (Raff et al., 1988). Astrocytes contribute to brain homeostasis, regulate synaptic plasticity, neurotransmission (Ferrer, 2017), and have long been considered crucial in sleep, and local blood flow (Ramon y Cajal, 1895; Howarth, 2014). Microglia are considered “surveillance cells” and are in charge of maintaining brain homeostasis, protecting against disease and infection (Graeber and Streit, 2010). They regulate CNS processing both via their connections with astrocytes and by interacting with synapses (Wake et al., 2009). Microglia can be protective by clearing cellular debris or deleterious by driving inflammation depending on their state of activation and the influence of steroid hormones (Larson, 2018).

Glia cells are also linked with steroid hormones. Oligodendrocytes secrete progesterone, and astrocytes secrete progesterone, testosterone, and estradiol-among other steroids (Gago et al., 2001; Garcia-Ovejero et al., 2005). Microglia have receptors for some of these steroids and are thus steroid-sensitive (Garcia-Ovejero et al., 2005). Recent evidence indicates that there might be more pronounced age-related changes in cell state, gene expression, and localization of glia cells than in neurons (Allen et al., 2023). Thus, the effects of steroid hormones on nonneuronal cells are of utmost importance to understand sex differences in aging. Sex differences in glia may affect the etiology of pathological conditions, and thus may also contribute to the possible sex differences in risk.

There are sex differences in macroglia. For instance, female rodents’ oligodendrocytes have a shorter lifespan than those in males but increased proliferation (Cerghet et al., 2006), and better migratory ability (Yasuda et al., 2020). With respect to neurodegenerative disease, an analysis of 80,660 single-nucleus transcriptomes from the postmortem prefrontal cortex of 48 individuals with AD revealed that higher AD pathology correlated with more global transcriptional activation in male oligodendrocytes than in female. Indeed, female oligodendrocyte precursors exhibited a down-regulation shift in response to pathology, a pattern that was not found in male cells (Mathys et al., 2019). These results offer new avenues to study myelination-related processes in AD pathogenesis. Given that E2 promotes oligodendrocytes myelin protein survival (Takao et al., 2004), these findings suggest that E2 play an important role in maintaining women’s white matter. Sex differences have also been shown for astrocyte function, number, and morphology (Sofroniew and Vinters, 2010; Chowen and Garcia-Segura, 2021). In non-human animal models, astrocyte proliferation is higher in females than in males (Llorente et al., 2009). Notably, estrogens acting on astrogliosis may lead to neural regeneration (Le Prince et al., 1993), and neuroprotection against AD (Liang et al., 2002). Progesterone may also provide neuroprotection against Aβ-induced neuroinflammation in astrocytes (Hong et al., 2016). A recent study has shown astrocytic dysfunction in AD (Serrano-Pozo et al., 2022), but the question of steroid hormones and sex differences related to astrocytes during AD awaits further research.

Microglial sex differences have been reported in number (Mouton et al., 2002), gene expression (Villa et al., 2019), and morphology (Lenz et al., 2013). Aging impacts microglia in that it affects gene expression and their reaction to stimulation (Chowen and Garcia-Segura, 2021). Critically, microglia have been suggested as the predominant cell to target AD pathology (Hu et al., 2023) and female microglia may age faster (Olmedillas del Moral et al., 2020). Greater expression of inflammation-related transcripts of microglia has been found in aged females than in males (Mangold et al., 2017). Moreover, microglia may contribute to the higher AD risk associated with *APOE ε4* in females (Stephen et al., 2019). In this respect, a recent study has shown an effect of *APOEε4* on medial temporal microglial activation leading to AD progression, this effect was independent of Aβ plaques and tau tangles (Ferrari-Souza et al., 2023). The study controlled for sex, but did not look at sex differences. This is important because their cohort of cognitively impaired individuals was 78% women, while the MCI individuals were almost 70% men. Finally, it has also been suggested that microglia are vulnerable to death by ferroptosis-a type of cell death that involves iron-dependent lipid peroxide accumulation (Dixon et al., 2012). Ryan et al. (2023) hypothesized that ferroptosis in microglia may drive neurodegeneration. Using a novel tri-culture system to create neurons, astrocytes and microglia, the authors found that microglia were the most responsive to iron and that ferroptosis in response to iron overload had a unique transcriptional signature found in neurodegeneration. These results are important because they may explain crucial mechanisms underlying neurodegenerative diseases. Indeed, E2 depletion in women after menopause may result in significant transcriptomic changes related to inflammation (Sárvári et al., 2012), and thus steroid action on microglia may explain the possible sex differences in AD.

### 3.2. Resilience in women

The concept of resilience is used to explain inter-individual variability in the trajectories of cognitive decline and AD (Stern, 2002). However, this term represents multiple concepts: cognitive reserve, brain reserve, compensation; and each of these concepts have different definitions. Here, we follow the current consensus framework and refer to resilience as a general concept that includes any term related to the ability of the brain to maintain cognition and function against normal or pathological aging (Stern et al., 2020). Following this approach, we focus on cognitive reserve as the flexibility of cognitive processes in the face of normal aging or disease, and brain reserve as the neurobiological state of the brain at any point in time: number of neurons, synapses, white matter integrity, etc. (Stern et al., 2020).

Indeed, much attention has been devoted in the last decades to studying resilience against AD and arrive at a consensus definition, but little attention has been devoted to elucidating the resilience proxies for women (Subramaniapillai et al., 2021) which might be different to those found in men. It is hypothesized that women may have had less access than men to some reserve-building factors such as education or work opportunities, and this, might in turn, lead to less cognitive reserve (Subramaniapillai et al., 2021). However, steroid hormones like estrogens and progesterone may affect brain plasticity and, in this way, contribute to brain reserve. A verbal advantage is also generally reported in women compared to males–even in MCI (Sundermann et al., 2016); thus, women’s verbal advantage can be considered a cognitive reserve proxy only among women. Crucially, the relationship between loss of estrogens and cognitive decline is a well-established phenomenon (Bove et al., 2014), and it may be the link needed to understand plasticity and resilience in older women.

#### 3.2.1. Neuroplasticity, steroid hormones, and brain reserve

Neuroplasticity is the result of functional and molecular changes within the CNS due to either experience/learning, injury, disease, or cell death (Hebb, 1949; Fuchs and Flügge, 2014). Steroid hormones play an essential role in neuroplasticity because they evoke changes in neuronal excitability that may induce synaptic plasticity (Baudry et al., 2013). Moreover, major hormonal shifts throughout the lifespan–puberty, pregnancy, menopause, and aging–typically trigger neuroplastic processes (Been et al., 2022). Aging is characterized by gonadal functional loss and is accompanied by subtle cognitive decline and frailty. These fluctuations of steroid hormones due to aging occur in both men and women, but the fluctuations in men are a gradual process and occur slowly with advancing age (Morley, 2003). In women, changes are more sudden, profound, drastic, and variable. For instance, motherhood is a period marked by deep neuroplastic changes (Pawluski et al., 2022; Orchard et al., 2023). Similarly, menopause may induce plasticity and neural reorganization (Jacobs and Goldstein, 2018); but there are several types of menopauses with the ovarian cycle ceasing for varied reasons – aging, ovarian surgery, cancer treatment–and at different ages (Edwards et al., 2019). Thus, it is important to also consider the reasons for menopause to determine the extent to which it induces neural plasticity. Despite this variability, neural plasticity – including regions that mediate memory–seems to be a constant in the lifespan of women (Sheppard et al., 2019). In humans, E2 shapes memory circuits in women by promoting hippocampal plasticity (Jacobs and Goldstein, 2018); in non-human animals it is been shown to control hippocampal and prefrontal cortical (PFC) dendritic spine proliferation (Hara et al., 2015). Indeed, the different type of estrogens may affect neuroplasticity and cognition in different ways; they may modulate hippocampal plasticity via cell proliferation and this effect is dependent on type, time and dose (Barha and Galea, 2010). Low levels of E2 may lead to better performance on spatial working memory and contextual fear conditioning, while high levels may impair spatial working memory, spatial reference memory, and contextual fear conditioning. Estrone also seems to impair contextual fear conditioning (Barha et al., 2010).

E2 has also been related to women’s PFC neural efficiency. For instance, in healthy young women, optimal cortical dopamine (DA) activity was linked to overall reduced PFC activity. However, PFC activity enhanced with greater demands for cognitive control in accordance with the neural efficiency hypothesis (Jacobs and D’Esposito, 2011). Thus, E2 may enhance cortical dopamine activity and alter PFC-dependent cognitive functions such as spatial memory and executive function. Moreover, E2 may be linked to hippocampal neurogenesis (Sager et al., 2018), essential to preserve cognition (Klempin and Kempermann, 2007). Importantly, in female rodents E2 seems to modulate neurogenesis while in male rodents it is androgens (Yagi and Galea, 2019). In addition, repeated administration of E2 to female rodents was related to increased cell proliferation and decreased cell death in the dentate gyrus (Barker and Galea, 2008). A transgenic AD mouse model showed that perturbed neurogenesis may result in new neurons having hyperphosphorylated tau, reduced hippocampal circuitry, and cognitive deficits like those seen in AD (Hollands et al., 2017).

Microglia cells show the highest level of CNS plasticity (Augusto-Oliveira et al., 2022). Of note, females have higher microglia density in the hippocampus during early development than males (Yanguas-Casás, 2020). In menopause, E2 depletion has a significant impact on microglia which results in an increased inflammatory phenotype (Yanguas-Casás, 2020). Interestingly, a recent study has shown that resilience is highly heritable in both sexes, but there might be sex specific genetic drivers of resilience. Genetic predisposition toward resilience may be linked to less genetic risk for autoimmune conditions among females (Eissman et al., 2022). In this sense, there is a dysregulation in immune response in AD pathogenesis, inflammatory process may play a primary role (Sardi et al., 2011), and E2 action in microglia may help elucidate the mechanisms underlying inflammation, AD and resilience in women.

The effects of progesterone on plasticity are not fully understood. Only a few studies have researched this question and the evidence shows contradictory results. One study showed that in CA1 slices from 4-week-old rats progesterone had no effect in long term potentiation (Ito et al., 1999), while others have shown an effect of progesterone on hippocampal synaptic transmission, likely mediated by GABA_A_ receptors (Foy et al., 2008). Progesterone may influence synaptic plasticity (Baudry et al., 2013), but its effects in resilience against AD for women are yet to be explored. As noted previously, interpretation about the effects of progesterone should carefully consider E2 activity. With mitochondria as a new perspective on women and AD (Nilsen and Brinton, 2004), a mouse model exploring the effects of progesterone and E2 on mitochondrial function is important. One study showed progesterone and E2 in opposition; both steroids improve mitochondrial function when administered separately but attenuate it when replaced in combination (Irwin et al., 2008).

Similarly, testosterone has been related to synaptic plasticity (Jia et al., 2016) but sex differences in resilience related to testosterone have not been fully explored. Benefits for men have been reported at the cognitive level. High testosterone may result in better long-term memory in men than in women (Barrett-Connor et al., 1999) and its decrease in men has been linked to poor performance in visual and verbal memory as well as visuospatial processing (Moffat et al., 2002). Thus, testosterone may also induce lifelong plasticity. However, the role of testosterone in cognitive decline for old adult men is not clear. In elderly men with low-normal gonadal status, a comprehensive neuropsychological evaluation showed no effect of low-normal testosterone status on cognition (Haren et al., 2005). A resilience effect of testosterone in women has been reported when analyzed in conjunction with genetic profile. A study of the 172 participants (113 men and 59 women) aged 55–90 years in the ADNI database showed that the effects of low testosterone are particularly detrimental to cognition in women who are APOE ε4carriers (Sundermann et al., 2020). The cohort included 15% cognitively normal participants, 56% with MCI and 23% with AD. In this case, the authors studied separate and interactive effects of testosterone levels and APOE ε4 on cerebrospinal fluid p-tau level. Sex difference in p-tau level was also considered before and after adjusting for testosterone. The results showed that women had higher p–tau levels than men among *APOE ε4* carriers, and this difference was eliminated when adjusting for testosterone. Thus, testosterone may induce plasticity in both men and women. Moreover, it may be protective against p-tau among women who are *APOE ε4* carriers. Importantly, women have typically less testosterone than men during the lifespan, and thus may be more at risk for AD due to higher levels of pathological Tau.

#### 3.2.2. Verbal cognitive reserve

Women have a verbal memory advantage as they age and as noted, this lasts into the pre-AD stage of MCI (Sundermann et al., 2016). The reasons for this are still unknown, but verbal memory in women is highly dependent on steroid hormone levels. Women show variations in verbal processing during pregnancy (Glynn, 2010), postpartum (de Groot et al., 2006), and menopause (Schaafsma et al., 2010). Fine motor coordination and verbal fluency depend on stage of the ovarian cycle with women at the early follicular (low estrogen and progesterone) performing worse than those at midluteal (high estrogen and progesterone; Maki et al., 2002). Mental rotation in women also depends on the menstrual cycle phase: mental rotation skills decreased during the midluteal phase but are on a par with men’s at early follicular (Hampson et al., 2014; Peragine et al., 2020). On the other hand, verbal fluency improves at high estradiol phases (Maki et al., 2002).

Verbal memory also improves in some postmenopausal women after taking estradiol hormone therapy (ET). Women taking estrogens had better verbal memory performance than those without (Kampen and Sherwin, 1994). This finding has been replicated in numerous studies (Sherwin, 1988; Jacobs et al., 1998; Wolf et al., 1999; Zec and Trivedi, 2002). Deficits in verbal memory are also reported in women after acute loss of ovarian function (Ryan et al., 2014; Soni and Hogervorst, 2014), and these effects can also be reversed with ET (Phillips and Sherwin, 1992; Newton et al., 1996; Sherwin and Tulandi, 1996; Craig et al., 2008). Similarly, verbal memory deficits have been reported in subjective cognitive decline which may lead to an increased risk for AD (Reuben et al., 2021). Attention and memory complaints of young women also vary across the menopausal transition. Data from 120 women who were either premenopausal, perimenopausal or postmenopausal showed attention complaints during perimenopause, and verbal memory problems directly related to SCD and menopause (Schaafsma et al., 2010). A study of SCD in a cohort of cognitively unimpaired individuals with autosomal-dominant AD who will develop dementia revealed that the women reported more SCD than men, and among female mutation carriers SCD was associated with worse verbal memory (Martinez et al., 2022).

The complaints in verbal memory among older women may be related to the loss of estradiol during menopause. However, once pathological cognitive decline begins, verbal memory may act as a form of reserve in some women. In an analysis of 742 participants with normal cognitive aging (NC), and early mild cognitive impairment (eMCI), the relationship between biological sex, verbal memory, hippocampal volume (HV) and florbetapir PET (an agent used to bind amyloid plaques for the potential detection of AD) showed that women with NC had a robust verbal advantage even in the face of amyloid positivity. On the other hand, women in the eMCI group, only showed decreased verbal memory in the face of amyloid positivity. The authors interpreted this finding as a verbal memory advantage for women with earlier stages of AD, but not in a stage of prodromal AD (Caldwell et al., 2017). Similarly, women with low to moderate Aβ burden (but not high Aβ burden) had increased verbal memory while men did not. Interestingly, this effect was specific to MCI (Sundermann et al., 2018). The relationship between biological sex, tau and verbal memory was explored in two different databases (National Alzheimer’s Coordinating Center, and the Alzheimer’s Disease Neuroimaging initiative) which revealed that in both, women had higher verbal reserve and were able to maintain this verbal reserve in the face of greater tau pathology than men (Digma et al., 2020). More recently, an analysis of sex differences in verbal memory and the distribution of tau pathology in MCI and AD patients showed a strong association between left hemisphere tau and verbal memory mainly in women with MCI (Banks et al., 2021).

The left hippocampus has been related to verbal memory, while the right hippocampus to spatial memory (de Toledo-Morrell et al., 2000). Both sides play a central role in the consolidation of memories and both suffer AD pathology. A study analyzing verbal memory in old adults found that the left hippocampal head may be the region directly associated with verbal memory (Hackert et al., 2002). E2 also modulates the metabolism of this as well as other brain regions (frontal, parietal, and temporal regions), and a few studies have shown that acute ovarian loss function may be linked to a decreased activation in the left inferior frontal gyrus (LIFG) during verbal memory encoding (Craig et al., 2009). Taken together, it’s important to consider that the maintenance of skills associated with the hippocampus and PFC such as verbal memory may be related to resilience in women.

#### 3.2.3. Sex differences and linguistic experiences

Given that there might be women’s verbal advantage linked to estradiol, leading to cognitive reserve, it is essential to analyze different linguistic experiences and how they may provide resilience to cognitive aging. For instance, multilingualism has also been suggested as a cognitive reserve proxy (Bialystok et al., 2007). Multilinguals with dementia can tolerate greater brain atrophy than monolinguals with similar clinical dementia levels (Schweizer et al., 2012). This pattern has been confirmed worldwide (Klein et al., 2016), and in different stages of neurodegeneration such as MCI (Duncan et al., 2018), conversion from MCI to AD (Calvo et al., 2023), and AD (Chertkow et al., 2010). A study of sex differences in bilingual and monolingual healthy old adults using the Wisconsin card sort test (WCST) revealed that women had more cognitive decline than men, but their performance improved with bilingualism (Subramaniapillai et al., 2019). Although the authors did not relate their finding to reserve, these results suggest a relationship between biological sex and multilingualism that may affect resilience mechanisms. These results should be taken with caution as the authors did not measure steroid hormones which is crucial for an analysis of this relationship.

Hearing loss is another linguistic experience worth considering to understand sex differences in risk of AD and plasticity (Bruce et al., 2019). Hearing impairment leads to problems in understanding spoken language and cognitive processing (Pichora-Fuller, 2003). Men are more affected by hearing loss than women in old age (Curhan et al., 2019) and recent studies indicate that estradiol may support the auditory system (Reavis et al., 2023). Indeed, a study investigating hearing threshold during the menstrual cycle found that the highest threshold in women is when estradiol levels are at its highest level (Da Da Souza et al., 2017). Moreover, premenopausal women have shown better extended high frequency hearing than postmenopausal women (Zhang et al., 2018), which emphasizes the need to consider estradiol effects in hearing loss or deafness in the elderly.

## 4. Discussion

The first person diagnosed with AD was a woman and also some of the most significant reports of resilience were done in cohorts including mostly women (Katzman et al., 1988; Snowdon, 1997). However, the higher risk of AD in women has only gradually come to recognition (Einstein, 1999). Historically, women have been excluded from clinical research because their biology was considered more complicated than that of men. This applied to both human and non-human animal research (Beery and Zucker, 2011; Shansky, 2019). In non-human animal models, female rats would typically be removed from experimentation because they were considered more “variable” and more expensive than males since they had to be tested at each stage of their estrous cycle. This assumption has been tested and refuted empirically (Prendergast et al., 2014). Nonetheless, most animal models of AD continue excluding females from their research (Beery and Zucker, 2011). In humans, policies have been implemented to ensure that women are included in clinical research. This has led to an expansion of research about women’s health and sex differences in health and disease. Importantly, more women are now enrolled in randomized clinical trials for AD than males which makes the analysis of sex differences in AD more feasible. However, only 12.5% of published articles have reported sex-stratified results (Martinkova et al., 2021). Thus, the full possibilities of sex differences in etiology, progression, treatment response, risk, and resilience in AD remain unexplored. Addressing sex differences in risk and resilience for AD is crucial for advancing successful interventions for the majority of the sufferers. By including empirical studies, reviews and meta-analyses looking at the effects of steroid hormones on aspects of AD and resilience, the current review is one step in that direction.

### 4.1. Neuronal effects

Our literature review indicated that steroid hormones could affect the etiology and progression of AD. Animal studies showed that estrogen deprivation results in spine density loss in crucial regions such as the hippocampus. In humans, it has been suggested that use of estrogens may decrease cognitive decline in postmenopausal women. The issue of HRT in AD is a critical one that has suffered from a scientific paradigm shift. Current evidence shows that the question of *when* HRT begins is crucial. Future research should also explore the benefits of ERT instead of CEE as most of the current evidence indicates that E2 loss after menopause may lead to AD. Importantly, the effects of E2 in women who have had BSO is a unique window to understand risk and resilience mechanisms in midlife.

Moreover, estradiol’s effect on hippocampal plasticity and neurogenesis (Jacobs and Goldstein, 2018; Yagi and Galea, 2019) may shape cognitive reserve in functions such as verbal memory (Sundermann et al., 2016; Digma et al., 2020). Progesterone may also have a beneficial effect but these positive effects may be lost when combined with estrogens (Pike et al., 2009). Testosterone may play a role in neurodegeneration in males; however, it may have a crucial role in guarding against p-tau in women who are *APOE ε4* carriers (Sundermann et al., 2018).

### 4.2. Non-neuronal effects

At the non-neuronal level, oligodendrocytes may aid resilience by protecting women’s white matter but the relationship with sex steroids has not yet been clarified. Astrocytes may depend on estradiol and progesterone for function and its presence may be related to resilience in AD (Liang et al., 2002; Serrano-Pozo et al., 2022), but no study has analyzed sex differences related to astrocytic function in AD. Microglia are more affected by aging in women than in men (Olmedillas del Moral et al., 2020). Microglia may also affect hippocampal neurogenesis (De Lucia et al., 2016). A few studies suggest that microglia may be associated with *APOE ε4* and higher risk for AD in women (Stephen et al., 2019). However, there is a dearth of studies that would illuminate the interaction of steroid hormones and microglia and its impact on microglial function in the etiology and progression of AD. This may be an important direction to pursue as inflammation is associated with AD, inflammation increases in menopausal women, and microglia play a role in brain inflammation. Thus, the effect of E2 decreases in microglia for postmenopausal women may help elucidate their role and the risks associated with inflammation in AD.

### 4.3. Factors leading to resilience

With regards to resilience, steroid hormones may impact both cognitive and brain reserve. For instance, verbal memory performance is one proxy of cognitive reserve for women. Brain regions crucial to verbal memory are the hippocampus, the PFC and the LIFG and they are all sensitive to E2. Indeed, E2 may be related to the neural efficiency of verbal memory in women (Jacobs and D’Esposito, 2011). Conversely, E2 loss has been associated with decreased activation in the LIFG during verbal memory encoding (Craig et al., 2009) as well as decrements in executive function and spatial memory performance, mediated by the PFC. Understanding the role of verbal memory as a reserve factor in women is crucial because most of the neuropsychological tests used to diagnose AD involve verbal processing. If verbal memory is, indeed, a cognitive reserve factor in women, and women have preserved verbal function compared to men, more sensitive tools may be needed to diagnose AD in women. Moreover, multilingualism should be considered as this has also been suggested as a proxy for cognitive reserve. Verbal memory and multilingualism may have an additive effect on cognitive reserve for women, and thus multilingual women may, in fact, be diagnosed so long into the disease that neural degeneration has progressed long past rescue. Further research is needed to understand the complex relationship between verbal memory and bilingualism in resilience for women. Importantly, studies assessing this question should focus on E2 effects in multilingual women to truly address reserve mechanisms in this cohort. Hearing loss is another linguistic experience that should be carefully considered. It seems to be more prevalent in men than in women (Curhan et al., 2019). Importantly, more men work in professions that affect hearing (e.g., armed forces; Thompson et al., 2016) and there is some thought that E2 is beneficial for maintaining the auditory system (Reavis et al., 2023). However, only a few studies are starting to explore these associations.

The analysis of estradiol on different linguistic experiences leads to reflection about other disorders in which language is affected. For instance, some neurodegenerative diseases are marked by language disturbances and thus the effects of estradiol in linguistic processing are crucial to understand the cause and course of these diseases. Consider the language variant of frontotemporal dementia (FTD) or primary progressive aphasia (PPAs). PPAs are typically sub-classified according to different linguistic criteria (Gorno-Tempini et al., 2011): a non-fluent/agrammatic variant (naPPA), a semantic variant (svPPA), and a logopenic variant (lvPPA). Few recent studies indicate sex differences in FTD in that women may be more affected by PPAs than men (Pengo et al., 2022). Indeed, women may have greater reserve to face FTD (Illán-Gala et al., 2021). However, to the best of our knowledge, no study has investigated estradiol effects in frontotemporal dementia. This is a critical new avenue to pursue as it may lead to the discovery of biomarkers that may help in the diagnosis and treatment of FTD as well as AD. It may also lead to the discovery of resilience mechanisms that are specific to FTD but not AD, and *vice-versa*.

### 4.4. Strengths and weaknesses

The strength of the current review is that it is synthetic, highlighting themes arising from the AD literature on the role of steroid hormones in AD mechanism as well as in risk and resilience. There are, however, some limitations. All women age and some experience severe drops in steroid hormone but only some of them get AD. The reasons for this are still unknown. It might be that AD pathology is dependent on levels of E2, years of E2 exposure or that E2 levels interacting with other biologically related variables – such as contraceptives, past hormone therapy, parity: affect AD pathology differently. Our review did not address this question, and future studies should include these variables to fully understand the role of E2 behind risk and resilience. Moreover, life circumstances such as access to education, complex occupation, high socio-economic standing, and misogyny are social factors embedded in gender which may also affect biology and hence, the risk of AD. We limited our question to biological sex and specifically pituitary-gonadal steroid hormone levels and thus, did not include studies analyzing the role of gender in AD. However, as noted, gender is crucial for the advancement of health equity. Another weakness is that we reviewed both human and non-human animal models and the ovarian cycle as well as menopause are different. Typically, women have a 30-day menstrual cycle and rodents have a 4–5-day estrous cycle. As well, rodents do not go into ‘menopause’; their aging ovarian state is either constant diestrous or constant estrous. Although the studies reviewed here suggest that E2 has a beneficial role in both humans and non-human animal modes, these differences in the estrous cycle and ovarian cessation should be considered carefully as they might have different impacts on cognitive decline.

## 5. Conclusion

Overall, the current review followed a synthetic approach to create a new theoretical perspective by rigorously analyzing patterns in the results of previous studies. We first explored new associations – steroid hormones, glia, AD–to identify how the loss of steroid hormones in aging may be related to risk in AD. We then investigated factors leading to AD resilience in women and identified new avenues for future research. Although more targeted studies are needed to understand these associations, the emerging themes summarized in this review highlight the importance of incorporating the analysis of steroid hormones in AD pathology. Specifically, the findings suggest that E2 loss after menopause may be one of the reasons for the increased risk of AD in women. Conversely, adequate levels of E2 are related to the brain and cognitive reserve mechanisms that may both lead to women’s unique pathways toward resilience against AD. One of the features of AD is cell death and E2 prevents neuronal death. It also shapes hippocampal plasticity, modulates neurogenesis and promotes neural efficiency in the PFC. Studying the effects of E2 on multiple brain systems can help to elucidate the complex nature of women’s risk and resilience and allow for the study of different types of reserve–brain and cognitive–in healthy or pathological aging. It may also help to identify new biomarkers with which to elucidate the risk, cause, trajectory, and resilience of women to many neurodegenerative diseases.

## Author contributions

All authors listed have made a substantial, direct, and intellectual contribution to the work and approved it for publication.

## Funding

This work was supported by the Wilfred and Joyce Posluns Chair in Women’s Brain Health and Aging from the Posluns Family Foundation, Canadian Institutes of Health Research (CIHR), Ontario Brain Institute, and Alzheimer Society of Canada to GE [grant WJP-150643], the Canadian Consortium on Neurodegeneration in Aging (CCNA) Phase II to GE (grant CCNA 049-04; CIHR reference number: CNA 163902), and the Jacqueline Ford Gender and Health Fund to GE.

## Conflict of interest

The authors declare that the research was conducted in the absence of any commercial or financial relationships that could be construed as a potential conflict of interest.

## Publisher’s note

All claims expressed in this article are solely those of the authors and do not necessarily represent those of their affiliated organizations, or those of the publisher, the editors and the reviewers. Any product that may be evaluated in this article, or claim that may be made by its manufacturer, is not guaranteed or endorsed by the publisher.
